# Evaluation of irisin and visfatin levels in very low birth weight preterm newborns compared to full term newborns—A prospective cohort study

**DOI:** 10.1371/journal.pone.0204835

**Published:** 2018-09-27

**Authors:** Nina Mól, Magdalena Zasada, Przemysław Tomasik, Katarzyna Klimasz, Przemko Kwinta

**Affiliations:** 1 Department of Paediatrics, Institute of Paediatrics, Faculty of Medicine, Jagiellonian University Medical College, Krakow, Poland; 2 Department of Clinical Biochemistry, Institute of Paediatrics, Faculty of Medicine, Jagiellonian University Medical College, Krakow, Poland; Medical University of Vienna, AUSTRIA

## Abstract

Premature infants represent one of the groups with increased risk for metabolic syndrome. Our study is the first one to evaluate irisin and visfatin levels, associated with the metabolic syndrome, both in blood of preterm and full-term infants, as well as in the breastmilk of their mothers. A total of 72 newborns was enrolled in the study, including 53 very low birth weight preterm infants and a control group of 19 term infants. The levels of irisin and visfatin were determined by a commercial enzyme-linked immunoabsorbent assay both in the baby serum and maternal milk twice, first during the 1^st^ week of life and then 4 weeks later. Preterm infants had significantly lower serum irisin levels compared to the term infants. Overall, serum irisin level during the 1^st^ week of life was positively correlated with several anthropometric measurements at birth, as well as during 5^th^ weeks of age. In contrast, serum visfatin levels during 5^th^ week of life were negatively correlated with z-scores of birth weight, weight and head circumference during 5^th^ week of age. We found a strong negative correlation between serum irisin and serum visfatin levels at both analyzed time points. The level of milk visfatin was significantly higher in the mothers of the preterm group during 5^th^ week of life. In conclusion, our results provide further evidence that irisin and visfatin may play physiologic roles in development of both preterm and full-term newborns during their first month after birth. Observed differences in irisin and visfatin serum and breastmilk concentrations during the earliest stages of life may contribute to development of catch up growth, but also, they might eventually lead to a higher risk for metabolic syndrome in prematurely born children in later years.

## Introduction

Preterm very low birth weight (VLBW) neonates are at a higher risk of metabolic syndrome later in life. Preterm infants at term equivalent age are lighter, shorter, have higher percentage of total fat mass and lower fat free mass [1], [2]. At 1–3 years, they exhibit excessive weight-for-length z-scores and elevated systolic blood pressure [3], [4]. Barker et al. shows a significant relationship between low birth weight, low weight at 1 year of life, catch up growth in childhood on one hand, and increased risk of adverse outcomes such as diabetes type 2, obesity, cardiovascular diseases on the other [5]. Numerous reports emphasize the importance of perinatal nutritional status for the survival of VLBW infants. It appears that childhood nutrition is critical in a regulation of lifelong appetite since it may affect some of the feeding regulatory mechanisms both in the central nervous system and peripheral tissues. Early life nutrition plays an important role in long-term appetite control. It is involved in the lifelong programming of feeding regulatory mechanisms in the central nervous system, including the ones mediated by factors from the peripheral tissues [6]. Early feeding model affects growth and body composition in the future [7].

Visfatin, originally identified as pre-B-cell colony-enhancing factor (PBEF), is a 491-amino-acid protein with a molecular weight 52 kDa [8]. It is produced and secreted from the visceral adipose tissue, which increase is strongly associated with the metabolic syndrome [9]. Visfatin is an adipokine that affects a number of metabolic and immune- processes, including regulation of white adipose tissue (WAT). It is up-regulated in obesity and insulin resistance [10], it exerts insulin like effects in various tissues [9]. Fukuhara et al. demonstrates a correlation between plasma visfatin concentrations and the amount of visceral fat [9]. There are studies that confirm significantly increased serum visfatin levels in neonates with intrauterine growth retardation (IUGR) and extremely low birth weight (ELBW) [11], [12].

Irisin is a newly discovered myokine with anti-obesity properties. Irisin regulates body energy expenditure by turning white adipose tissue into brown-like adipose tissue [13]. It is regarded as a potential biomarker of metabolic syndrome and obesity. In clinical settings, patients with type 2 diabetes mellitus have lower levels of irisin compared to healthy controls [14], [15]. Moreover, irisin’s precursor, fibronectin type III domain-containing protein 5 (FNDC5), is decreased in patients with obesity [16].

Visfatin and irisin are also present in human milk. It is suggested that autonomous production by breast tissue is the source of those hormones in the breast milk. It appears that these bioactive peptides found in the breast milk are important for growth, energy regulation and maturation of the gastrointestinal system in neonates [17], [18], [19]. The physiologic role of irisin and visfatin in neonates, especially in VLBW infants, remains to be studied. The goal of our prospective study was to analyze concentrations of irisin and visfatin both in the VLBW and full-term infants’ serum, as well as their mothers’ breast milk. We also examined an association between blood irisin and visfatin levels and selected anthropometric parameters.

## Materials and methods

### Study design and area

The prospective observational study included newborns and their lactating mothers admitted to the Neonatal Intensive Care Unit, Department of Paediatrics, Jagiellonian University Medical College, Krakow, Poland, between February 2014 and November 2016. The study protocol was approved by the Jagiellonian University Medical College Ethical Committee (issue No KBET/58/B/2013 from 4.04.2013). Written and informed consents were obtained from the parents. Study was sponsored by NUTRICIA Foundation (RG 7/2013).

### Inclusion criteria

Study group consisted of preterm newborns with birth weight between 1000 -1500g and term newborns with birth weight between 2500g - 4000g. All the neonates admitted to the hospital who fulfilled inclusion criteria (admission before 5^th^ day of life, feeding started within first 48 hours of life) were included in the study. Only 2 children were not enrolled due to lack of the parental consent. Full term newborns were admitted to the NICU due to moderate ailments that frequently occurred in the neonatal period (e.g. severe hyperbilirubinemia, transient tachypnoea of newborn or other adaptation disorders), which did not affect proper physical development in researcher’s opinion. A formal sample size calculation was not performed to allow a realization of a hypothesis generating study. The approximate sample size was based on the calculations from the previous publication [20]. The power analysis [21] indicated that with 53 experimental subjects and 19 control subjects, the estimated power of the study to validate the measured means difference of the fat mass at the level of 100g was 0.8 (p = 0.05). Using mentioned above method we performed the power calculation for the present study. The estimated detectable true difference between the means of serum irisin concentration was 0.6 ug/ml, and between the means of serum visfatin concentration was 1.6 ng/ml.

### Exclusion criteria

Exclusion criteria for all the groups were: severe congenital malformations, chromosomal aberrations, asphyxia (5^th^ minute Apgar Score < 3 points), intraventricular hemorrhage grade IV, and severe infections (early onset neonatal sepsis, meningitis).

All infants required parenteral infusion of glucose, lipids and amino acids from the time of their admission until their oral feeding reached adequate volumes. All infants started oral feeding within 24–48 hours with either breast milk, which was supplemented with a standard dose of human milk fortifier when the feeding portion exceeded 140 mls/kg/day (Bebilon HMF, Nutricia), or a formula specially adapted for the preterm infant (PreNAN, Nestle, or Bebilon Nenatal, Nutricia). The infants were fed by nipples or nasogastric tubes. Each mother was encouraged to provide milk for her own infant; due to lack of availability we could not use human milk from a milk bank.

### Anthropometric measurements

Anthropometric measurements such as weight, length, and head circumference were collected twice: at birth, and then 4 weeks later (during 5^th^ week of life). All infants were weighed naked to the nearest 10g on an electronic baby scale (RADWAG 2006). Crown-heel length and occipito-frontal circumference were measured to the nearest 0.5cm by a standard measuring tape. A single investigator made all the measurements. Anthropometric parameters Z-score were obtained using a reference by Fenton et al. [22]. The BIA analysis to determine body composition was carried out either at estimated time of birth in the VLBW groups or during the 1st week of life in the control group. The data regarding body composition were presented in the results section. Detailed description of BIA analysis was provided elsewhere [20].

### Hormone assays

The samples were collected twice: during the 1^st^ week of life, and then 4 weeks later (5^th^ week of life). We used test tubes with aprotinin, a protease inhibitor (Becton Dickinson Vacutainer) to collect blood and milk samples. Blood samples were transferred immediately after drawing to the Department of Clinical Biochemistry, Institute of Pediatrics, Jagiellonian University Medical College, Krakow, Poland and centrifuged. The supernatant plasma was separated and immediately stored in a deep freezer (-80°C) until assay. Milk samples were transferred to the above laboratory and immediately stored in a deep freezer (-80°C) until assay.

Levels of irisin were measured by an ELISA commercial kit (RAG018R, BioVendor, Brno, Czech Republic). The minimum detectable concentration, intraassay and interassay coefficients of variation were 0.001μg/ml, 5–8%, 8–10%, respectively.

Levels of visfatin were measured by an ELISA commercial kit (RAG004R, BioVendor, Brno, Czech Republic). The minimum detectable concentration, intraassay and interassay coefficients of variation were 0.003μg/ml, 2.5–9%, 4.66–7.24%, respectively.

### Statistical analysis

JMP 13.1.0 software (SAS Institute, Cary, North Carolina) was used for statistical analysis. Demographic and clinical data comparisons between the study and control groups were performed using Student’s t-test or chi- square test, depending on the data character and their distribution. The data were expressed as arithmetic means ± SD. The detected differences were considered statistically significant in case of p<0.05. Correlations between hormone levels and anthropometric measurements were analysed using Spearman’s test.

## Results

The baseline characteristics of the study population were summarized in Table 1. The baseline characteristics of the patients’ mothers, including possible causes of preterm birth were shown in Table 2. We found no differences between mothers of VLBW and full-term infants, except thyroid disease frequency that was significantly higher in the mothers of preterm babies.

**Table 1:** Baseline characteristics of the study and control populations. Data are presented as mean with standard deviation (SD), or number and fraction of patients (%) as appropriate.

	Study group VLBW infants (n = 53)	Control group term infants (n = 19)	p-value
Gestational age [weeks], mean (SD)	30 (2.03)	39 (1.32)	<0.0001^1^*
Birth weight [g], mean (SD)	1257.0 (161.8)	3396.4 (421.6)	<0.0001^1^*
Birth weight z-score, mean (SD)	-0.28 (0.94)	0.1 (0.89)	0.12^1^
Birth length [cm], mean (SD)	40.02 (2.6)	53.5 (3.63)	<0.0001^1^*
Birth length z-score, mean (SD)	0.48 (0.95)	1.99 (0.92)	<0.0001^1^*
Birth head circumference [cm], mean (SD)	27.14 (1.59)	34.4 (1.26)	<0.0001^1^*
Birth head circumference z-score, mean (SD)	-0.05 (1.22)	0.05 (1.07)	0.753^1^
Weight at 4^th^ weeks [g], mean (SD)	1707.27 (277.22)	4026 (461.31)	<0.0001^1^*
Weight at 4^th^ weeks z-score, mean (SD)	-0.93 (0.82)	1.32 (0.83)	<0.0001^1^*
Length at 4^th^ weeks [cm], mean (SD)	43.71 (2.41)	55.8 (3.19)	<0.0001^1^*
Length at 4^th^ weeks z-score, mean (SD)	0.07 (0.90)	3.19 (1.69)	<0.0001^1^*
Head circumference at 4^th^ weeks [cm], mean (SD)	29.37 (1.50)	36.63 (1.40)	<0.0001^1^*
Head circumference at 4^th^ weeks z-score, mean (SD)	-0.65 (0.79)	1.79 (1.08)	<0.0001^1^*
Male gender	28 (53%)	13 (68%)	0.239^2^
Vaginal delivery	10 (19%)	7 (37%)	0.113^2^
Small for Gestational Age	6 (11%)	0 (0%)	0.126^2^

P-value was significant in case of *p<0.05.

1—Student’s t-test

2—chi-square test.

**Table 2:** Maternal characteristics of the study population. Data are presented as mean with standard deviation (SD), or number and fraction of patients (%) as appropriate.

	Study group VLBW infants (n = 53)	Control group term infants (n = 19)	p
Maternal age [years], mean (SD)	29.15 (5.49)	30.84 (5.11)	0.2335^1^
Gestational weight gain [kg], mean (SD)	9.58 (5.50)	12.18 (4.86)	0.0643^1^
Gestation			0.3155^2^
1	26 (49%)	5 (26%)	
2	15 (28%)	10 (53%)	
≥3	12 (23%)	4 (21%)	
Pregnancy complications			
Hypertension	14 (26.4%)	2 (10.5%)	0.1301^2^
Diabetes	4 (7.5%)	1 (5.3%)	0.7156^2^
Thyroid disease	6 (11.3%)	7 (36.8%)	0.0184^2^*

P-value was significant in case of *p<0.05.

1 –two-sided T-test

2—chi-square test.

Preterm VLBW infants at their expected time of birth had higher fat mass and percentage amount of fat mass, whereas their total body water, percentage amount of TBW and percentage amount of FFM were lower compared to the full-term control group during their 1^st^ week of life (Table 3).

**Table 3:** Body composition of the study population presented as mean values and SD.

	Study group VLBW infants (n = 53)	Control group term infants (n = 19)	P
TBW [kg]	2.57 (0.46)	3.15(0.69)	0.0017^1^*
TBW %	72.05 (7.71)	92.78 (14.16)	0.0001^1^*
FFM [kg]	2.99 (0.47)	2.88 (0.35)	0.3685^2^
FFM %	83.71 (1.67)	85.49 (1.31)	0.0001^2^*
FM [kg]	0.59 (0.16)	0.49 (0.1)	0.0275^1^*
FM %	16.29 (1.67)	14.51 (1.31)	0.0001^2^*

P-value was significant in case of *p<0.05.

1 –Wilcoxon/Kruskal-Wallis test

2 –two-sided T-test.

TBW- total body water, TBW %- percentage amount of total body water, FFM- fat free mass, FFM %- percentage amount of fat free mass, FM- fat mass, FM %- percentage amount of fat mass.

**Fig 1:**
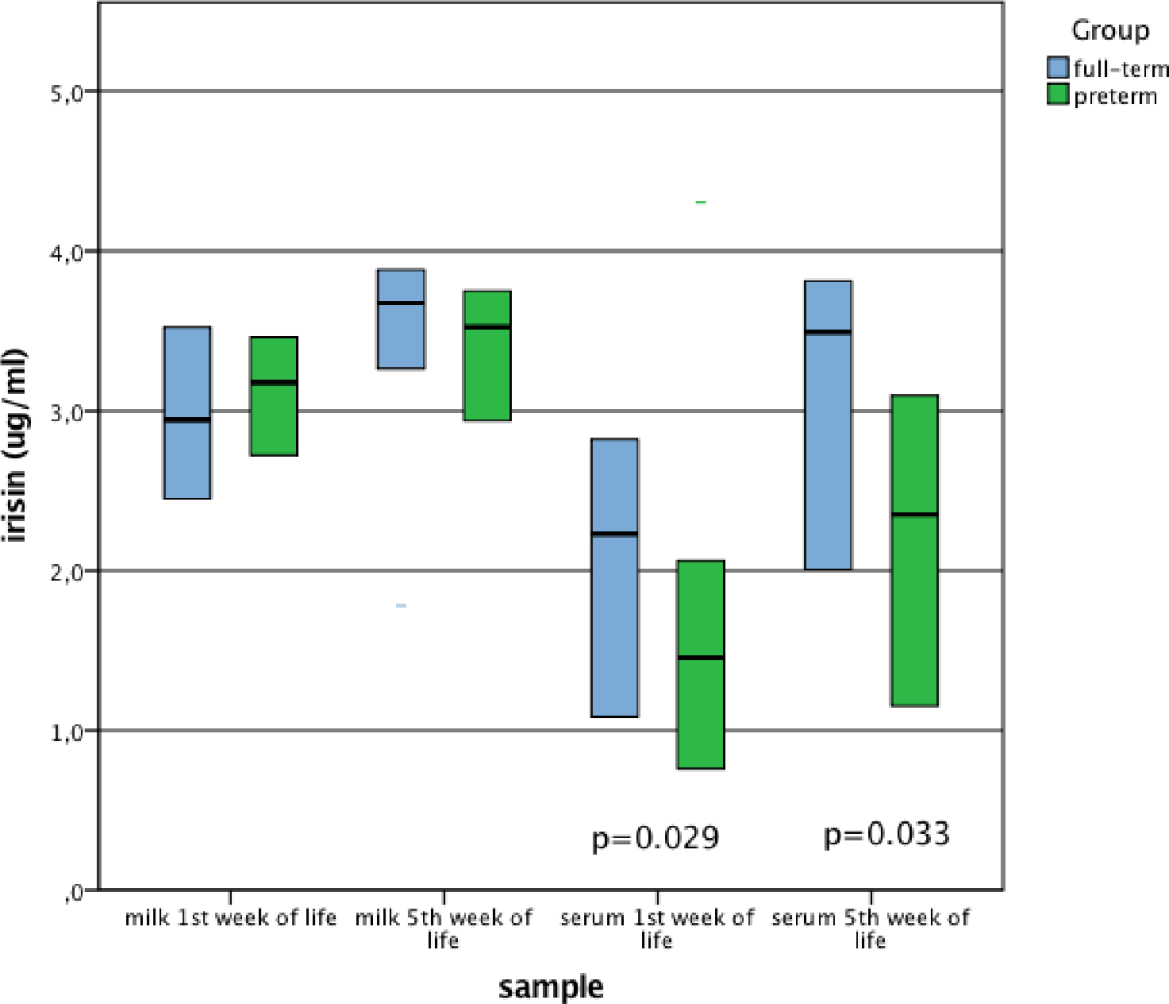
Comparison of milk and serum irisin concentrations in the groups of full-term and preterm newborns. Data are presented as median and interquartile range, P-values for U Mann-Whitney test.

**Fig 2:**
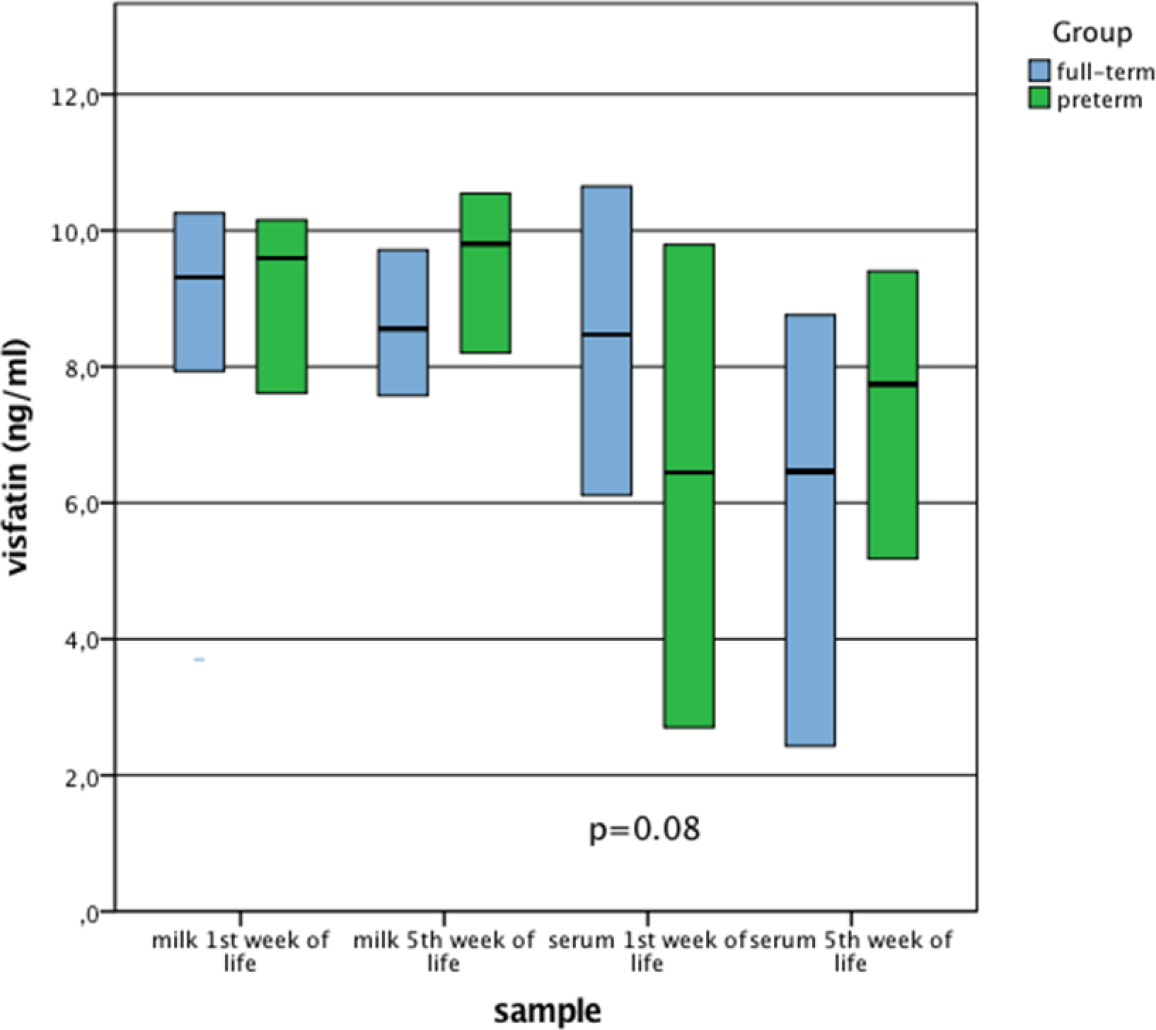
Comparison of milk and serum visfatin concentrations in the groups of full-term and preterm newborns. Data are presented as median and interquartile range, P-value for U Mann-Whitney test.

Preterm VLBW infants had significantly lower serum irisin levels compared to the term infants both during the 1^st^ and 5^th^ week of life. Milk visfatin level in the study group increased over the span of 4 weeks, and it was significantly higher in the 5^th^ week of life. The level of milk visfatin was significantly higher in the mothers of the preterm group during 5th week of life. There were no other significant differences between serum visfatin levels, milk visfatin and milk irisin levels in both time points (Figs 1 and 2).

Serum irisin level during the 1^st^ week of life was positively correlated with several anthropometric parameters including birth weight, birth length, head circumference at birth, weight and head circumference at 5 weeks. Serum irisin levels during the 1^st^ week of life were positively correlated with their concentration after 4 weeks. Serum visfatin levels in the second sampling, at 5^th^ week of life, were negatively correlated with z-scores for birth weight, length and head circumference. Interestingly, we found a strong negative correlation between serum irisin and serum visfatin during the 1^st^ week of life, which persisted over the following 4 weeks of the study (Table 4).

**Table 4:** Spearman’s correlation between serum and milk irisin and visfatin levels and anthropometric parameters in the two tested groups.

Variable	by Variable	Spearman ρ	Prob>|ρ|
Serum irisin levels during 1^st^ week (μg/ml)	Birth weight (g)	0.3768	0.0011
	Birth length (cm)	0.3300	0.0046
	Birth head circumference (cm)	0.2562	0.0298
	Weight at 4^th^ week (g)	0.3160	0.0110
	Head circumference at 4th weeks (cm)	0.2690	0.0316
Serum irisin levels during 5^th^ week (μg/ml)	Serum irisin levels during 1^st^ week	0.4293	0.0005
Serum visfatin levels during 1^st^ week (ng/ml)	Serum irisin levels during 1^st^ week	-0.3612	0.0018
Serum visfatin levels during 5^th^ week (ng/ml)	Serum irisin levels during 5^th^ week	-0.5318	<0.0001
Serum visfatin levels during 5^th^ week (ng/ml)	Birth weight z-score	-0.2576	0.0433
	Weight at 4^th^ week z-score	-0.3071	0.0152
Milk irisin levels during 5^th^ week (μg/ml)	Milk irisin levels during 1^st^ week	0.5664	0.0006
Milk visfatin levels during 5^th^ week (ng/ml)	Milk visfatin levels during 1^st^ week	0.7071	<0.0001

Milk irisin during the 1^st^ week of life was positively correlated with milk irisin level 4 weeks later. Milk visfatin during the 1^st^ week of life was also positively correlated with milk visfatin level at the second time point of the study (Table 4).

We found some differences in the levels of irisin and visfatin in preterm babies’ mothers between the ones with and without pregnancy complications (data not shown). Mothers diagnosed with hypertension had significantly higher breastmilk irisin levels than mothers without hypertension during 5^th^ week of infants’ life. In case of diabetes mellitus, the mothers presented significantly lower serum visfatin levels during 1^st^ week after birth and significantly higher visfatin levels 4 weeks later compared to the mothers without diabetes mellitus.

## Discussion

Our prospective cohort study shows that blood irisin levels are positively correlated with weight, length and head circumference of a child at birth, as well as with his weight and head circumference 4 weeks later. It has been reported that cord blood irisin levels are positively correlated with birth weight [23], [24], birth weight Z-scores and gestational age [25]. Further, irisin level in the umbilical artery is positively correlated with fetal weight and fetal abdominal circumference as measured by ultrasonography [26]. Our findings support and expand the above observations, since the samples were collected from the newborns during their 1^st^ and 5^th^ week of life. Our study confirms that VLBW preterm infants present significantly lower serum irisin levels compared to the term infants during the 1^st^ and 5^th^ week of life, and there is a positive correlation between first and second assessment. Decreased irisin might result in smaller amount of fat free mass and higher fat mass observed in VLBW group at estimated time of birth. To our best knowledge this is the first report of irisin blood levels in VLBW newborns. Studies from the literature show that infants born as small for gestational age (SGA) present decreased levels of irisin in comparison to their appropriate for gestational age (AGA) counterparts [23], [24], [25], which might result from their much smaller muscular mass and reduced brown adipose tissue [24], [25], [27]. Previous studies in full term infants demonstrate irisin levels overall higher compared to the findings in our study [24]. We suppose that we detect lower irisin levels since our blood samples are collected directly from the infants, in contrast to the previous studies that presented irisin analyses in the umbilical blood most probably affected by the maternal serum irisin. We do not observe any significant differences in serum visfatin levels between VLBW and term newborns during their 1^st^ week and 5^th^ week of life. Our findings are similar to those observed by Cekmez et al, [12], however they contradict other reports demonstrating lower serum visfatin in VLBW infants compared to the full-term infants [11], [28] and adults [29]. In our study serum visfatin levels during 5^th^ week of life are negatively correlated with the birth weight z-score, weight z-score at 5 weeks, and head circumference z-score at 5 weeks in contrast to some previous studies [28]. We assume that the observed relative decrease of visfatin might be associated with the rapid catch-up growth observed in VLBW and ELBW newborns [30], [31].

We find a strong negative correlation between serum irisin and serum visfatin levels during 1^st^ as well as 5^th^ week of life. The above hormones present antagonizing actions, since irisin shows anti-obesity properties while visfatin increases in obesity. It is possible that if one of these hormone increases, then the other one is down-regulated, although the exact molecular mechanisms remain to be discovered.

There is a number of reports demonstrating that human milk contains a variety of hormones which regulate body weight and composition [17], [32], [33], [34], [35]. One of them is irisin, however its role in regulation of newborn development is to be discovered. Aydin et al. find that levels of irisin are highest in colostrum, then decrease in transitional and mature milk from healthy women. It is different in gestational diabetes, where irisin is significantly lower in the colostrum and transitional milk than in the mature milk [18]. The highest irisin concentration is found in maternal blood, even compared to colostrum as reported by Brianna and colleagues [36]. Our study is the first one to assess irisin and visfatin levels in breast milk of mothers of VLBW infants. We find no differences between irisin levels measured in milk samples from mothers of VLBW infants compared to samples from mothers of term newborns. We hypothesize that breast milk irisin levels may affect postnatal adaptation with respect to thermoregulation, glucose metabolism and neonatal homeostasis, which further supports introduction of early breastfeeding.

In the early studies visfatin was identified in the mammalian epithelial cells in the mammary gland and milk, later confirmed in the breast milk in humans, reaching significantly higher concentrations than in the maternal serum [37][17]. Moreover, visfatin concentration in colostrum could be used for prediction of the subsequent weight development of the infant, demonstrated as less or more severe weight loss during the first 3 days of life [17]. This observation strongly suggests that increased concentration of visfatin in the human milk plays a protects of against a weight loss during its first days after birth. Bienertová-Vašků et al. speculate that the above effects might result from breast milk visfatin- mediated insulin-like effect on child’s adipose tissue [17]. In contrast to the previous studies, we found increased visfatin levels in breast milk during 5^th^ week postpartum in VLBW group. Since VLBW infants are much more susceptible to severe weight loss after birth, we believe that significantly higher visfatin levels in their mother’s milk may represent some additional, natural protection against it.

In conclusion, the results of our study suggest that irisin and visfatin may represent a potential link between VLBW status at birth and later metabolic syndrome; however, a direct causal relationship cannot yet be confirmed. Such a hypothesis needs to be addressed in a longitudinal study of irisin levels, postnatal weight gain and their association with obesity and metabolic syndrome in the future.
